# Artificial intelligence‐based analysis of body composition predicts outcome in patients receiving long‐term mechanical circulatory support

**DOI:** 10.1002/jcsm.13402

**Published:** 2023-12-26

**Authors:** Isabell Anna Just, Felix Schoenrath, Luise Roehrich, Emanuel Heil, Julia Stein, Timo Alexander Auer, Uli Fehrenbach, Evgenij Potapov, Natalia Solowjowa, Felix Balzer, Dominik Geisel, Juergen Braun, Georg Boening

**Affiliations:** ^1^ Department of Cardiothoracic and Vascular Surgery German Heart Center Berlin Berlin Germany; ^2^ DZHK (German Centre for Cardiovascular Research), Partner Site Berlin Berlin Germany; ^3^ Department of Cardiothoracic Surgery Charité – Universitätsmedizin Berlin Berlin Germany; ^4^ German Heart Foundation Frankfurt am Main Germany; ^5^ Department of Cardiology German Heart Center Berlin Berlin Germany; ^6^ Department of Radiology Charité – Universitätsmedizin Berlin, corporate member of Freie Universität Berlin, Humboldt‐Universität zu Berlin, and Berlin Institute of Health Berlin Germany; ^7^ Berlin Institute of Health (BIH) Berlin Germany; ^8^ Institute of Medical Informatics Charité – Universitätsmedizin Berlin, corporate member of Freie Universität Berlin, Humboldt‐Universität zu Berlin, and Berlin Institute of Health Berlin Germany

**Keywords:** artificial intelligence, body composition, cachexia, left ventricular assist device, obesity paradox, sarcopenia

## Abstract

**Background:**

Obesity is a known cardiovascular risk factor and associated with higher postoperative complication rates in patients undergoing cardiac surgery. In heart failure (HF), conflicting evidence in terms of survival has been reported, whereas sarcopenia is associated with poor prognosis. An increasing number of HF patients require left ventricular assist device (LVAD) implantations. The postoperative mortality has improved in recent years but is still relatively high. The impact of body composition on outcome in this population remains unclear. The aim of this investigation was to examine the preoperative computed tomography (CT) body composition as a predictor of the postoperative outcome in advanced HF patients, who receive LVAD implantations.

**Methods:**

Preoperative CT scans of 137 patients who received LVADs between 2015 and 2020 were retrospectively analysed using an artificial intelligence (AI)‐powered automated software tool based on a convolutional neural network, U‐net, developed for image segmentation (Visage Version 7.1, Visage Imaging GmbH, Berlin, Germany). Assessment of body composition included visceral and subcutaneous adipose tissue areas (VAT and SAT), psoas and total abdominal muscle areas and sarcopenia (defined by lumbar skeletal muscle indexes). The body composition parameters were correlated with postoperative major complication rates, survival and postoperative 6‐min walk distance (6MWD) and quality of life (QoL).

**Results:**

The mean age of patients was 58.21 ± 11.9 years; 122 (89.1%) were male. Most patients had severe HF requiring inotropes (Interagency Registry for Mechanically Assisted Circulatory Support [INTERMACS] profile I–III, 71.9%) secondary to coronary artery diseases or dilated cardiomyopathy (96.4%). Forty‐four (32.1%) patients were obese (body mass index ≥ 30 kg/m^2^), 96 (70.1%) were sarcopene and 19 (13.9%) were sarcopene obese. Adipose tissue was associated with a significantly higher risk of postoperative infections (VAT 172.23 cm^2^ [54.96, 288.32 cm^2^] vs. 124.04 cm^2^ [56.57, 186.25 cm^2^], *P* = 0.022) and in‐hospital mortality (VAT 168.11 cm^2^ [134.19, 285.27 cm^2^] vs. 135.42 cm^2^ [49.44, 227.91 cm^2^], *P* = 0.033; SAT 227.28 cm^2^ [139.38, 304.35 cm^2^] vs. 173.81 cm^2^ [97.65, 254.16 cm^2^], *P* = 0.009). Obese patients showed no improvement of 6MWD and QoL within 6 months postoperatively (obese: +0.94 ± 161.44 months, *P* = 0.982; non‐obese: +166.90 ± 139.00 months, *P* < 0.000; obese: +0.088 ± 0.421, *P* = 0.376; non‐obese: +0.199 ± 0.324, *P* = 0.002, respectively). Sarcopenia did not influence the postoperative outcome and survival within 1 year after LVAD implantation.

**Conclusions:**

Preoperative AI‐based CT body composition identifies patients with poor outcome after LVAD implantation. Greater adipose tissue areas are associated with an increased risk for postoperative infections, in‐hospital mortality and impaired 6MWD and QoL within 6 months postoperatively.

## Introduction

Obesity is a common comorbidity of patients undergoing cardiac surgery. Multiple studies reported an association with higher rates of wound infections and sternal dehiscences promoted by more complex anatomies, deeper surgical wound surfaces and concomitant diabetes mellitus.^1^, ^2^, ^3^ Furthermore, obesity was identified as a risk factor for early postoperative atrial fibrillation^4^ and acute renal failure.^5^ However, data on perioperative mortality are conflicting, so that currently, it remains unclear whether an obesity paradox, referring to beneficial outcomes in obese patients compared with non‐obese patients, applies to this cohort.^6^, ^7^, ^8^, ^9^, ^10^

Left ventricular assist device (LVAD) implantation is a standard surgical treatment for advanced heart failure (HF). Even though postoperative in‐hospital mortality has improved in recent years, it remains high, compared with other cardiac surgical procedures.^11^ Patient selection is becoming increasingly important to identify candidates with manageable perioperative risk profiles who will prognostically truly benefit from an LVAD.

In this context, body composition has been evaluated as a risk factor for postoperative outcome after LVAD implantation.^12^ In patients with higher body mass indexes (BMIs) at implantation, more device‐related infections, pump thrombosis, HF hospital admissions and neurological complications were observed.^13^, ^14^, ^15^ In a large single‐centre analysis of 618 patients receiving an LVAD, a BMI > 30 kg/m^2^ was associated with an increase in mid‐term mortality.^16^ However, most authors reported no impact on survival.^14^, ^15^

The BMI is known to have various deficiencies as a measure of obesity, as it is not accurately accounting for body fat, which applies in particular to men, elderly and individuals with intermediate BMI ranges.^17^ Few studies have been performed analysing sarcopenia measured by computed tomography (CT)‐derived muscle areas or bioelectrical impedance analysis (BIA), indicating an impaired outcome after LVAD implantation in sarcopenic HF patients.^18^, ^19^, ^20^, ^21^ However, in these studies, adipose tissue has not been sufficiently considered as a possible confounder.

A combined investigation of the muscle and adipose body composition is feasible by analysing CT scans, and since its introduction as an imaging biomarker, a correlation with prognosis was shown in a variety of non‐HF cohorts.^22^, ^23^, ^24^ Additionally, artificial intelligence (AI)‐based analyses were shown to be superior to manual analyses of single CT slices.^22^, ^23^, ^24^, ^25^, ^26^

The aim of this work was to evaluate the AI‐based CT body composition parameters as predictors of postoperative outcome of patients undergoing LVAD implantation.

## Methods

### Study design

We conducted a post‐analysis of the FrailtyVAD‐Tx study cohort (NCT04222400) to evaluate the association of AI‐based CT body composition parameters and postoperative outcome of patients undergoing contemporary continuous‐flow LVAD implantation at the German Heart Center Berlin between January 2017 and December 2019. The analysis was approved by the local ethics committee of the Charité – Universitätsmedizin Berlin (EA2/236/17).

### Study individuals and data collection

We screened data of 169 adult patients of the FrailtyVAD‐Tx study who underwent LVAD implantations at our institution within the investigational period. Thirty‐two patients were excluded in whom no preoperative abdominal CT scan was available within 60 days prior to LVAD implantation. A total number of 137 patients were found eligible and were included in the final analysis. The following data were gathered and extracted as available: age; BMI; LVAD type; Interagency Registry for Mechanically Assisted Circulatory Support (INTERMACS) profiles; preoperative diagnosis, haemodynamics and functional capacity; comorbidities and body composition; postoperative days in intensive care unit (ICU) and in hospital; complications within 30 days; re‐hospitalizations within 6 months; functional capacity and quality of life after 6 and 12 months; and in‐hospital and 12‐month survival (*Figure* *S1*).

### Functional testing and quality of life assessments

As part of the FrailtyVAD‐Tx trial, all mobilized patients performed 6‐min walk tests preoperatively and 12 months postoperatively according to standards of the American Thoracic and European Respiratory Society.^27^ For quality of life assessments, the five‐level version of the EQ‐5D questionnaire was used.^28^

### Computed tomography scans

CT scans were obtained from preoperative routine diagnostics and had to include the third lumbar vertebral body for analysis. Median time from CT scan to LVAD implantation was 7 days [interquartile range, IQR, 2, 20 days]. If more than one CT was available, the one with the shortest time to the LVAD implantation date was chosen. Patients were examined on several high‐end CT scanners following different contrast standard protocols representing clinical daily practice.

### Artificial intelligence‐based computed tomography body composition

For the analysis of body composition, we used an AI‐based automated software tool based on a convolutional neural network, U‐net, developed for image segmentation (Visage Version 7.1, Visage Imaging GmbH, Berlin, Germany) as described before.^23^ The network automatically identified axial CT images of the L3 level in thick slices of the latest contrast phase. The image identification was manually controlled by an experienced radiologist (>10 years of abdominal reading). Tissue was automatically separated into psoas muscle, skeletal muscle, visceral fat and subcutaneous fat and coded with different colours. Other tissues, such as kidney, liver, spleen, intestine and pancreas, were not segmented. False tissue segmentation occurred in three cases, for example, when hypodense stool in the intestine was misinterpreted as body fat and was manually corrected. Other corrections were necessary due to the presence of material artefacts. The discrepancy of the results between AI and manual segmentation was in a very low percentage range as described in a previous work.^29^

**Figure 1:**
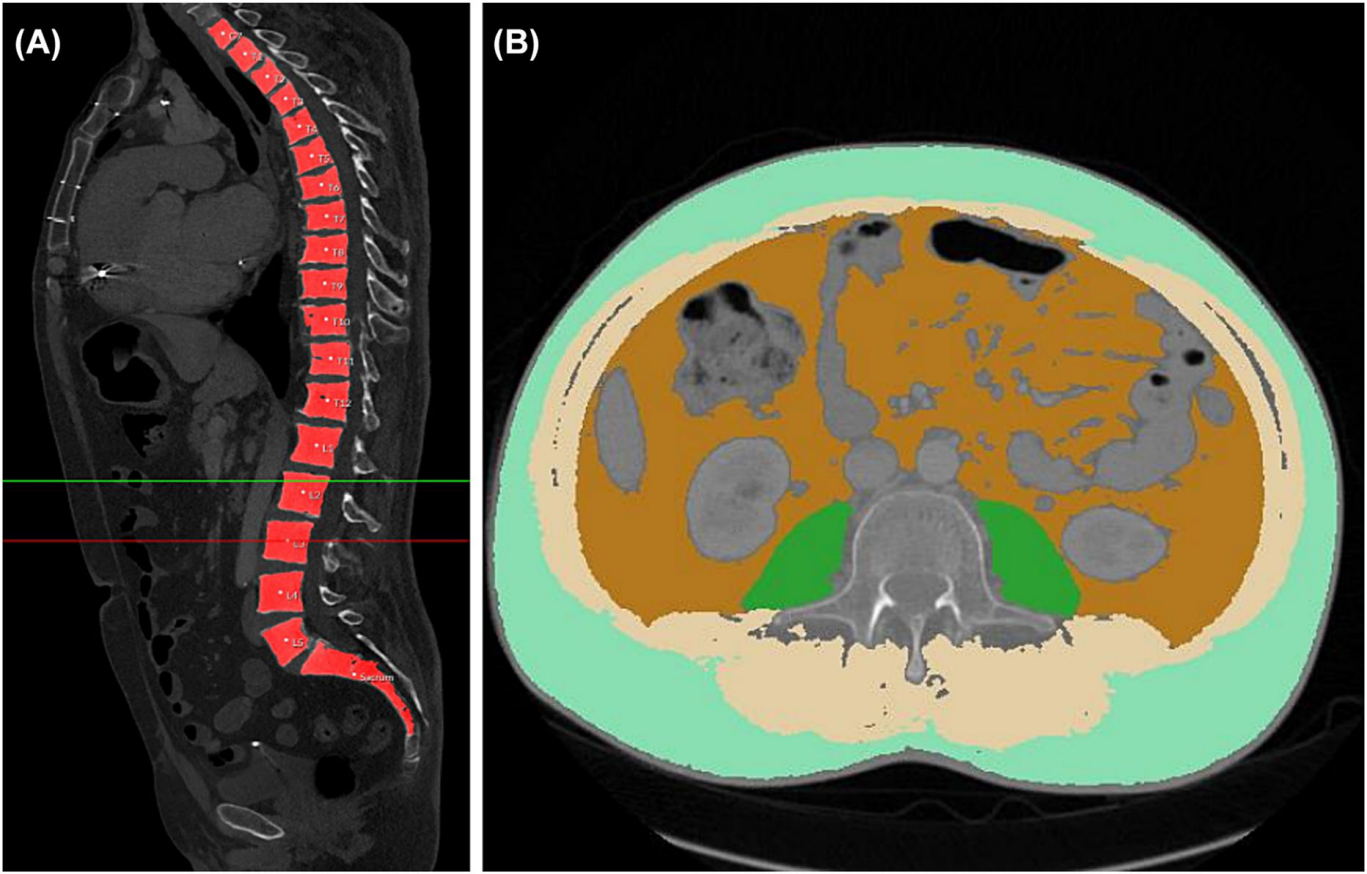
Example of the detection of the measuring plane (red line) on mid‐vertebral level L3 (A). Example of the artificial intelligence‐based segmentation of subcutaneous adipose tissue, visceral adipose tissue and psoas muscle (B).

The area in square centimetres and density in Hounsfield units of each segmented tissue class were automatically calculated by the software. The following parameters were derived from L3 body composition analysis: mean density (in Hounsfield units) of skeletal muscle including the psoas muscle, and areas (in square centimetres) of skeletal muscle, visceral adipose tissue (VAT), subcutaneous adipose tissue (SAT), psoas muscle area (PMA) and total abdominal muscle area (TAMA). The abdominal adipose tissue ratio (ATR) was calculated as the quotient of VAT and SAT (VAT/SAT); obesity was defined as BMI ≥ 30 kg/m^2^; and sarcopenia was defined as lumbar skeletal muscle index (LSMI) values of ≤38.5 cm^2^/m^2^ for women and ≤52.4 cm^2^/m^2^ for men, as described previously.^26^ Sarcopenic obesity was defined as both sarcopenic and obese. An example of AI‐based automated analysis of L3 body composition is shown in *Figure* *1*.

### Statistical analysis

Categorical data were summarized as frequencies and percentages. Continuous data were summarized as mean and standard deviation (SD) or in the case of skewed data, as median and IQR. The *χ* ^2^ test, Mann–Whitney *U* test or Student's *t*‐test were used for comparison as appropriate. Preoperative/postoperative analyses were performed using Wilcoxon's tests. Spearman's test or linear regression analysis was performed to explore the correlations as appropriate to analyse the effect of body composition parameters on outcome variables. SPSS (Version 25, IBM Corp., Chicago, USA) was used for statistical analysis.

## Results

### Baseline characteristics

One hundred thirty‐seven patients were included in the analysis. The mean age was 58.21 ± 11.86 years; 89.1% were male. LVAD implantation was mainly (96.4%) performed for the treatment of dilated cardiomyopathy or ischaemic heart disease. The mean left ventricular ejection fraction (LVEF) at implantation was 18.85 ± 6.32%; the right ventricular ejection fraction (RVEF) was 41.02 ± 9.03%. Most patients (90.5%) were implanted in acute HF INTERMACS profile I–IV with a median N‐terminal prohormone of brain natriuretic peptide (NT‐proBNP) level of 7056.0 pg/mL [IQR 3585.0, 15 363.0 pg/mL]. Baseline characteristics of all patients are summarized in *Table* *1*.

**Table 1:** Preoperative patient characteristics at left ventricular assist device implantation

	Total *n* = 137	Missing (%)
Sex (%)		0
Male	122 (89.1)	
Age in years	58.21 ± 11.86	0
Diagnosis (%)		0
DCM	60 (43.8)	
IHD	72 (52.6)	
Others^a^	5 (3.6)	
Comorbidities (%)		0
Diabetes mellitus	26 (19.0)	
COPD	23 (16.8)	0
LVEF in %	18.85 ± 6.32	0
LVEDD in mm	66.56 ± 11.46	0
RVEF in %	41.02 ± 9.03	0
VAD type (%)		0
HM3	38 (27.7)	
HVAD	98 (72.3)	
INTERMACS profile (%)		0
I	24 (18.8)	
II	43 (33.6)	
III	25 (19.5)	
IV	32 (25.0)	
>IV	5 (3.6)	
MCS (%)		0
ECMO	12 (8.8)	
Miniature axial flow pump	9 (6.6)	
IABP	1 (0.7)	
RRT (%) biomarkers	13 (9.6)	0
NT‐proBNP, pg/mL	7056.0 [3585.0, 15 363.0]	12.4
LDH, U/L	272.0 [217.5, 358.5]	3.6
Creatinine, mg/dL	1.5 [1.0, 1.8]	9.6
γGT, U/L	119.0 [69.75, 231.25]	1.4
Lactate, mmol/L	8.0 [6.0, 12.0]	2.1

*Note*: Continuous data were expressed as mean (± standard deviation); categorical data were expressed as number (percentage); and skewed data were expressed as median [interquartile range]. Abbreviations: COPD, chronic obstructive pulmonary disease; DCM, dilated cardiomyopathy; ECMO, extracorporeal membrane oxygenation; HM3, HeartMate 3; HVAD, HeartWare assist device; IABP, intra‐aortic balloon pump; IHD, ischaemic heart disease; INTERMACS, Interagency Registry for Mechanically Assisted Circulatory Support; LDH, lactate dehydrogenase; LVEDD, left ventricular end‐diastolic diameter; LVEF, left ventricular ejection fraction; MCS, mechanical circulatory support; NT‐proBNP, N‐terminal prohormone of brain natriuretic peptide; RRT, renal replacement therapy; RVEF, right ventricular ejection fraction; VAD, ventricular assist device; γGT, γ‐glutamyltransferase.

^a^ Other: valvular heart disease (*n* = 2), congenital heart disease (*n* = 2) and hypertrophic cardiomyopathy (*n* = 1).

### Body composition

Mean BMI was 28.21 ± 5.93 kg/m^2^; 32.1% of patients were obese. A total of 70.1% of patients were sarcopene and 24.8% sarcopene and obese. AI‐based CT body composition is summarized in *Table* *2* (and an additional presentation of data by sex is available in *Table* *S1*).

**Table 2:** Preoperative body composition

	Total *n* = 137
BMI in kg/m^2^	28.21 ± 5.93
BMI ≥ 30 kg/m^2^ (%)	44 (32.1)
VAT in cm^2^	159.96 ± 115.61
SAT in cm^2^	200.67 ± 118.97
ATR	0.82 ± 0.46
PMA in cm^2^	17.76 ± 4.99
TAMA in cm^2^	143.55 ± 31.45
LSMI in cm^2^/m^2^	46.28 ± 9.97
Sarcopenia (%)	96 (70.1)
Sarcopenic obesity (%)	34 (24.8)

*Note*: Continuous data were expressed as mean (± standard deviation); categorical data were expressed as number (percentage); and skewed data were expressed as median [interquartile range]. Abbreviations: ATR, abdominal adipose tissue ratio; BMI, body mass index; LSMI, lumbar skeletal muscle index; PMA, psoas muscle area; SAT, subcutaneous adipose tissue; TAMA, total abdominal muscle area; VAT, visceral adipose tissue.

### Preoperative risk factors

There was no association of body composition biomarkers and most known preoperative risk factors for impaired postoperative outcome including RVEF, NT‐pro BNP levels, preoperative 6‐min walk distance (6MWD) or preoperative quality of life. The ATR was higher in patients with creatinine levels > 1.2 mg/dL (*Table* *S2*).

### Body composition and postoperative complications

Within 30 days, postoperative infections requiring antibiotic treatment occurred in 70 (51.1%) patients. Patients who developed an infection had significantly higher preoperative adipose tissue areas than patients without postoperative infections (VAT 172.23 cm^2^ [IQR 54.96, 288.32 cm^2^] vs. VAT 124.04 cm^2^ [IQR 56.57, 186.25 cm^2^], *P* = 0.022, respectively). A preoperative VAT ≥ 200 cm^2^ was associated with a 2.6‐fold elevated risk for the development of a postoperative infection (odds ratio [OR] 2.627, 95% confidence interval [CI] 1.231–5.606, *P* = 0.011). The risk for postoperative infections increased to 4.2‐fold in sarcopene obese patients (OR 4.227, 95% CI 1.324–13.500, *P* = 0.010).

Preoperative adipose tissue was not associated with the occurrence of postoperative right HF, need for renal replacement therapy or strokes within 30 days after LVAD implantation (*Table* *3*). None of the muscle tissue imaging biomarkers (sarcopenia, PMA, TAMA and LSMI) were associated with postoperative complications (*Table* *3*).

**Table 3:** Body composition and postoperative outcome

Outcome		Adipose tissue			Muscle tissue			Sarcopene		Sarcopene obese	
		VAT	SAT	ATR	PMA	TAMA	LSMI	Yes	No	Yes	No
Infection	Yes	172.23 [54.96, 288.32]	198.84 [117.23, 306.61]	0.81 [0.53, 1.17]	15.99 [14.16, 19.47]	141.52 [127.74, 166.76]	45.63 [40.59, 53.34]	46 (48.4)	24 (58.5)	15 (78.9)	55 (47.0)
	70 (51.1)										
	No	124.04 [56.57, 186.25]	169.50 [110.98, 243.86]	0.67 [0.43, 1.07]	18.29 [14.03, 21.76]	134.73 [121.48, 160.25]	44.63 [38.56, 51.84]	59 (51.6)	17 (41.5)	4 (21.1)	62 (53.0)
	66 (48.2)										
	*P*‐value	0.022	0.052	0.223	0.119	0.202	0.367	0.279		0.010	
RRT	Yes	161.86 [96.16, 246.94]	185.29 [114.66, 271.60]	0.80 [0.55, 1.00]	16.80 [14.62, 19.55]	141.70 [130.24, 171.23]	44.72 [40.08, 59.11]	14 (14.7)	7 (17.1)	3 (15.8)	18 (15.4)
	21 (15.3)										
	No	151.60 [54.05, 233.68]	223.90 [119.00, 335.87]	0.73 [0.45, 1.11]	16.85 [13.91, 20.87]	137.41 [122.11, 161.46]	45.20 [39.57, 52.37]	81 (85.3)	34 (82.9)	16 (84.2)	99 (84.6)
	115 (83.9)										
	*P*‐value	0.397	0.090	0.798	0.909	0.239	0.658	0.729		0.964	
Right heart failure	Yes	162.16 [153.38, 228.72]	227.28 [148.36, 318.24]	0.81 [0.64, 0.91]	16.80 [13.80, 19.54]	135.28 [130.69, 149.96]	45.20 [41.43, 48.16]	4 (4.2)	3 (7.3)	1 (5.3)	6 (5.1)
	7 (5.1)										
	No	149.21 [54.91, 239.06]	185.29 [113.21, 270.20]	0.73 [0.45, 1.10]	16.85 [14.13, 20.70]	137.92 [122.41, 167.08]	45.04 [39.47, 53.02]	91 (95.8)	38 (92.7)	18 (94.7)	111 (94.9)
	129 (94.2)										
	*P*‐value	0.439	0.327	0.949	0.836	0.996	0.973	0.452		0.980	
Stroke	Yes	101.78 [67.71, 140.93]	190.14 [117.02, 243.86]	0.60 [0.38, 0.91]	16.98 [13.82, 19.55]	130.64 [104.04, 148.59]	45.22 [37.87, 48.07]	7 (7.4)	1 (2.4)	0 (0)	8 (6.8)
	8 (5.8)										
	No	155.75 [53.61, 246.82]	188.91 [111.90, 281.94]	0.75 [0.46, 1.12]	16.84 [14.02, 20.78]	138.54 [125.09, 167.93]	45.12 [39.55, 53.12]	88 (92.8)	40 (97.6)	19 (100)	109 (93.2)
	128 (93.4)										
	*P*‐value	0.189	0.904	0.296	0.168	0.824	0.542	0.262		0.240	

Abbreviations: ATR, abdominal adipose tissue ratio; LSMI, lumbar skeletal muscle index; PMA, psoas muscle area; RRT, renal replacement therapy; SAT, subcutaneous adipose tissue; TAMA, total abdominal muscle area; VAT, visceral adipose tissue.

### Body composition and postoperative outcome

The median stay on the ICU was 15 days [IQR 6.00, 34.25 days], and the overall hospital stay was 45 days [IQR 26.25, 75 days]. The preoperative body composition was not associated with a prolonged length of hospital stay or ICU stay (*Figure* *S1*).

**Figure 2:**
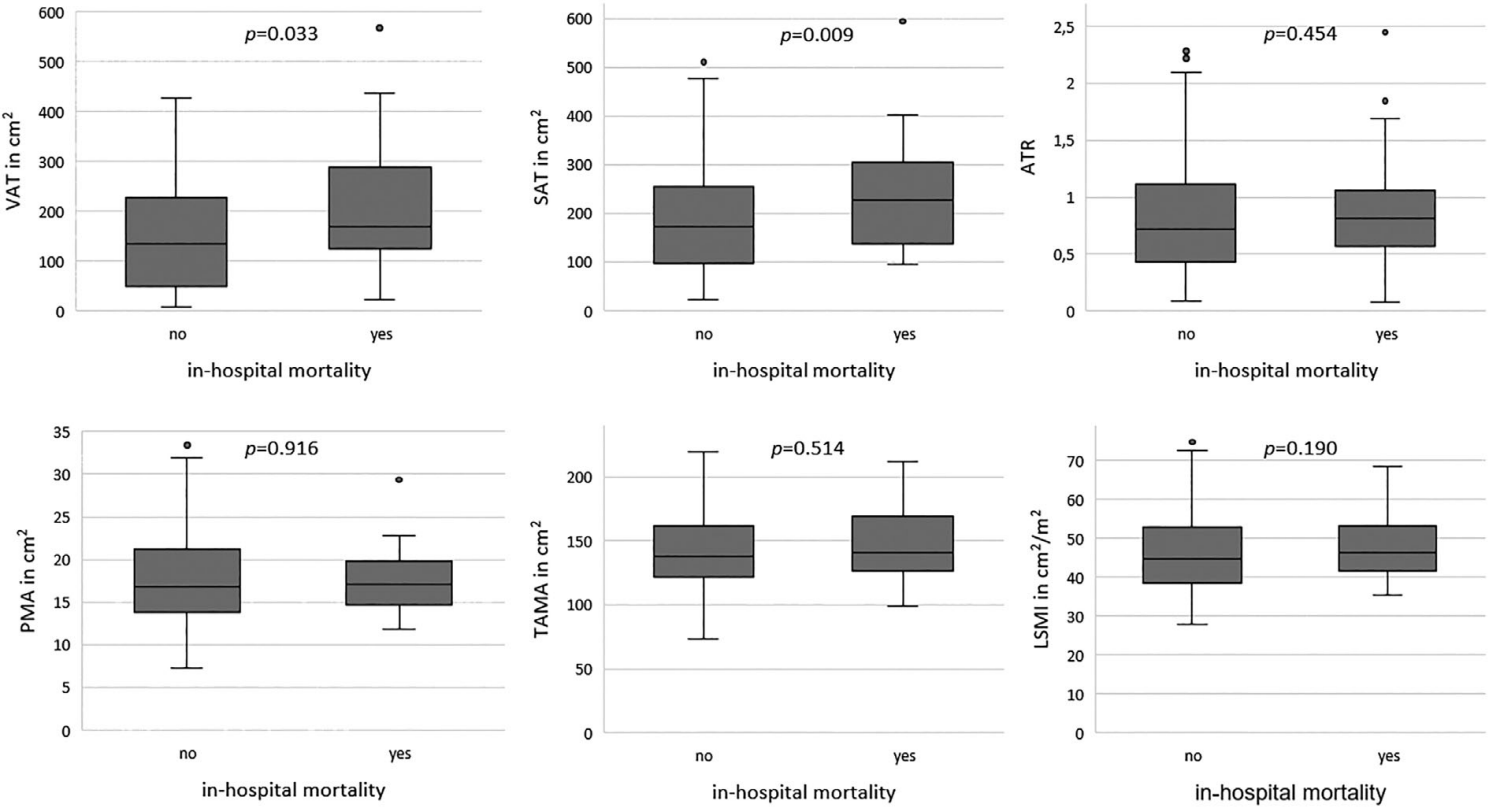
Boxplots of preoperative body composition by in‐hospital mortality after left ventricular assist device implantation. ATR, abdominal adipose tissue ratio; LSMI, lumbar skeletal muscle index; PMA, psoas muscle area; SAT, subcutaneous adipose tissue; TAMA, total abdominal muscle area; VAT, visceral adipose tissue.

Twenty‐nine (21.2%) patients died within the hospital stay after median 24 days [10, 69 days]. Cause of death was sepsis in 15 (10.9%) patients, neurological events including intracranial bleeding (*n* = 3, 2.2%) and stroke (*n* = 4, 2.9%), and prolonged cardiogenic shock in 4 (2.9%) patients, haemorrhagic shock (*n* = 1, 0.7%) and right HF (*n* = 1, 0.7%). Patients who died within the hospital stay had significantly higher preoperative VAT areas and SAT areas than patients who survived the initial hospital stay (VAT 168.11 [134.19, 285.27] vs. 135.42 [49.44, 227.91], *P* = 0.033; SAT 227.28 [139.38, 304.35] vs. 173.81 [97.65, 254.16], *P* = 0.009, respectively). Preoperative muscle biomarkers showed no impact on short‐term or 12‐month survival after LVAD implantation (*Table* *S3* and *Figure* *2*).

**Figure 3:**
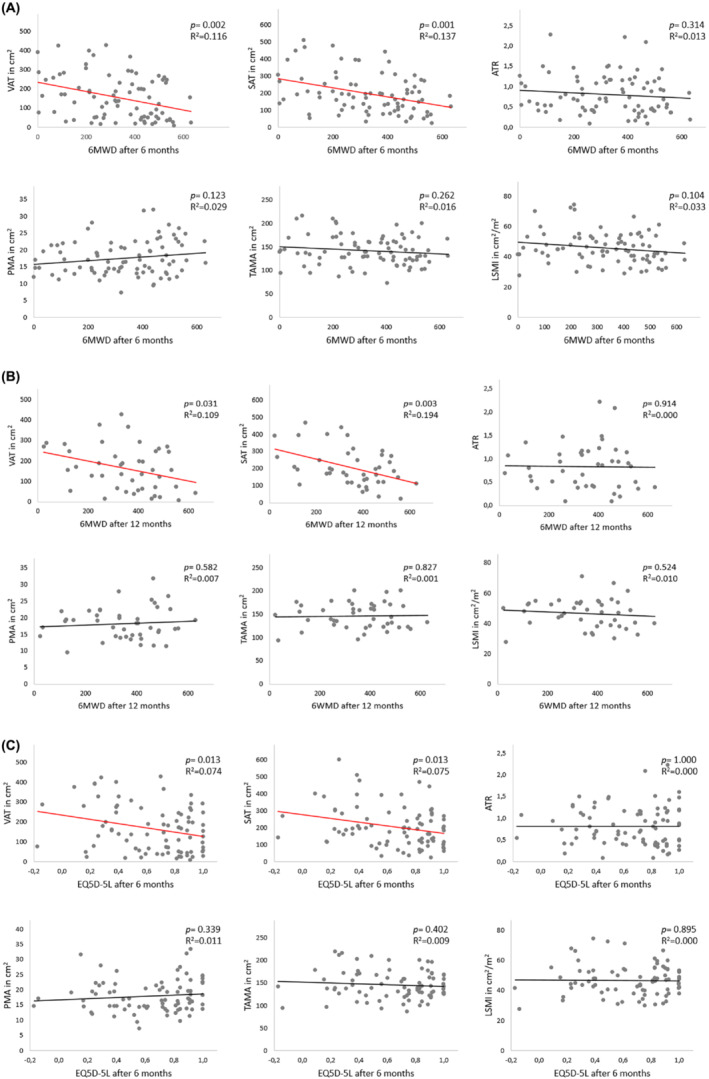
Correlation analysis and linear curve fitting. Body composition and 6‐min walk distance (6MWD) after 6 months (A) and 6MWD after 12 months (B) as well as 6 months of quality of life (C) after left ventricular assist device implantation. ATR, abdominal adipose tissue ratio; LSMI, lumbar skeletal muscle index; PMA, psoas muscle area; SAT, subcutaneous adipose tissue; TAMA, total abdominal muscle area; VAT, visceral adipose tissue.

Of the preoperative body composition biomarkers, only preoperative VAT areas and SAT areas correlated with absolute postoperative 6MWD at 6 months (available for *n* = 81 patients) and at 12 months (available for *n* = 43 patients) after LVAD implantation. VAT and SAT biomarkers exclusively correlated with the quality of life 6 months after implantation (available for *n* = 82 patients). The less the VAT areas and SAT areas, the better the 6MWD and quality of life (*Figure* *3*).

The 6MWD before LVAD implantation did not differ in patients irrespectively of the presence of obesity, sarcopenia or sarcopenic obesity. The 6MWD 6 months after implantation was significantly higher exclusively in patients who were non‐obese preoperatively when compared with preoperatively obese patients (370.19 ± 150.995 vs. 251.28 ± 166.00, *P* = 0.002; *Table* *S4*).

In 36 patients, 6MWD was available both before and 6 months after LVAD implantation (baseline characteristics of this subgroup are presented in *Table* *S5*). Of these, preoperatively non‐obese patients, patients with a preoperative VAT area < 200 cm^2^ or non‐sarcopene obese patients had a significant increase in 6MWD 6 months after LVAD implantation (+166.9 ± 139.0 months, *P* < 0.001; +144.90 ± 145.51, *P* < 0.001; and +123.59 ± 149.64, *P* < 0.001, respectively; *Table* *4*). Obese individuals or patients with a preoperative VAT area ≥ 200 cm^2^ had no increase in 6MWD 6 months postoperatively. Preoperatively, sarcopene patients showed a significant improvement in 6MWD, whereas sarcopene obese patients or non‐sarcopene patients showed no improvement (*Table* *4*). The postoperative 6MWD did not differ between sarcopene or sarcopene obese patients when compared with non‐sarcopene or non‐sarcopene obese patients (sarcopene: 0.520 ± 0.346 vs. non‐sarcopene: 0.418 ± 0.247, *P* = 0.240; sarcopene obese: 250.09 ± 153.80 vs. non‐sarcopene obese: 346.93 ± 162.91, *P* = 0.068).

**Table 4:** Change in body composition and 6‐min walk distance or quality of life 6 months after left ventricular assist device implantation

	∆ 6MWD preoperative to after 6 months in months	*P*‐value
Non‐obese	+166.90 ± 139.00	0.000
Obese	+0.94 ± 161.44	0.959
VAT < 200 cm^2^	+144.90 ± 145.51	0.000
VAT ≥ 200 cm^2^	+28.44 ± 178.88	0.569
Non‐sarcopene	+48.08 ± 120.06	0.224
Sarcopene	+115.67 ± 187.41	0.005
Non‐sarcopene obese	+123.59 ± 149.64	0.000
Sarcopene obese	−33.00 ± 199.02	0.735

	∆ QoL preoperative/after 6 months	*P*‐value
Non‐obese	+0.199 ± 0.324	0.001
Obese	+0.088 ± 0.421	0.500
VAT < 200 cm^2^	+0.192 ± 0.349	0.008
VAT ≥ 200 cm^2^	+0.109 ± 0.387	0.211
Non‐sarcopene	+0.226 ± 0.313	0.015
Sarcopene	+0.121 ± 0.387	0.066
Non‐sarcopene obese	+0.184 ± 0.320	0.001
Sarcopene obese	−0.013 ± 0.571	0.866

*Note*: Data are presented as mean ± standard deviation. Abbreviations: 6MWD, 6‐min walk distance; QoL, quality of life; VAT, visceral adipose tissue area.

The quality of life before LVAD implantation did not differ in patients irrespectively of the presence of obesity, sarcopenia or sarcopenic obesity. Preoperative and 6 months postoperative assessments were available in 50 patients (baseline characteristics of this subgroup are presented in *Table* *S5*). Of these, 19 (%) patients were obese, 21 (%) had a VAT area ≥ 200 cm^2^, 33 (66%) were sarcopene and 7 (14%) were sarcopene and obese. The postoperative quality of life was significantly better in patients who were non‐obese when compared with obese patients (non‐obese: 0.695 ± 0.275 vs. obese: 0.560 ± 0.311, *P* = 0.049; *Table* *S4*). A significant improvement of quality of life occurred exclusively in patients without obesity, sarcopenia or sarcopenic obesity and in patients with a VAT < 200 cm^2^ (*Table* *4*).

## Discussion

An obesity paradox, describing the relationship between obesity and a favourable disease outcome compared with normal‐weight individuals, has been described for various entities including HF.^8^, ^9^, ^30^ In patients requiring cardiac surgery, the obesity paradox has been discussed controversially. Our LVAD cohort represents the combined constellation of a severe HF population requiring cardiac surgery.

In our analysis, the AI‐based evaluation of the preoperative body composition demonstrated that adipose tissue was associated with an increased risk for postoperative complications and in‐hospital mortality. The exercise capacity and quality of life remained impaired over at least 6 months postoperatively in obese individuals with VAT ≥ 200 cm^2^. Single muscle tissue imaging biomarkers were not associated with an impaired outcome.

Obesity was shown to increase the rates of complications and mortality after cardiac surgery, and it has been referred to as the only modifiable preoperative risk factor for the development of a mediastinitis.^1^, ^2^, ^31^, ^32^ In line with this, in our cohort, obese patients (i.e., higher BMI or higher VAT levels) had an increased risk for infections. In general, postoperative infections represent severe complications, but particularly in LVAD patients, septic infections carry the risk for LVAD‐associated endoplastitis, which are extremely challenging in terms of pathogen cultivation and treatment due to biofilm formation and therefore represent a devastating adverse event.^33^, ^34^

Our patients with higher VAT or SAT imaging biomarkers had a worse postoperative in‐hospital survival. Patients mainly died due to septic complications, which was in line with the significantly higher infection rates. Overall, the in‐hospital mortality rate of 21% was higher than reported by the European Registry for Patients with Mechanical Circulatory Support (EUROMACS) registry (era 2014–2016: 17.5%; era 2017–2020: 11.2%).^11^ However, compared with the registry cohort, our patients presented more often in critical cardiogenic shock or progressive haemodynamic decline (INTERMACS level I and II in era 2014–2017: 15.8% and 30.7%, era 2018–2020: 13.5% and 25.6% vs. our cohort: 18.8% and 33.6%, respectively).^11^ Furthermore, the definition of in‐hospital mortality might have been used inconsistently, that is, when patients are transferred to other hospitals, as the 90‐day mortality rate in our cohort (19.0%) was comparable with the previously reported 90‐day mortality rate of 20% from a EUROMACS cohort.^33^

In our analysis, the 1‐year survival was not affected by the preoperative body composition. However, this does not exclude an impact of body composition on outcome in LVAD patients in general, as changes in adipose and muscle tissue composition might occur postoperatively, potentially affecting the long‐term outcome, as a recent study performed by Vest and colleagues showed fat mass (further) increase after LVAD implantation.^35^ Moreover, this might in particular affect the long‐term outcome of younger patients, in whom obesity influences the eligibility for heart transplantation. Especially in transplant regions with high scarcity of donor organs, obesity represents a contraindication for transplantation in order to insure best possible graft survival.

The available literature on body composition and post‐cardiac surgery outcome is inconsistent. In a large meta‐analysis from Mariscalco and colleagues, as well as in a study from a US database including more than 6 200 000 patients, a lower risk in obese patients was reported after cardiac surgery.^7^ However, in these analyses, a minority of patients (12% and 1%, respectively) had (severe) HF. Furthermore, as in most studies, the BMI was used as a measure of obesity without considering the composition of adipose and muscle tissue.

In our cohort, preoperative muscle biomarkers did not show an impact on the rate of adverse events, the length of ICU stay or length of hospital stay and mortality after LVAD implantation. The impact of sarcopenia in HF patients before LVAD implantation has been investigated by other groups with conflicting results: Sarcopenia was often associated with an increased length of hospital stay and increased early mortality rates.^18^, ^19^, ^21^, ^36^ Other studies showed no survival benefit in non‐sarcopenic patients,^20^, ^37^ as in our cohort. Overall, the comparability of these studies is highly limited as data were derived from different cohorts; for example, in one study, only patients on non‐contemporary HeartMate II devices (Abbott, Abbott Park, IL, USA) were investigated. Additionally, measurements of muscle mass were performed by CT or BIA. CT‐derived muscle mass referred to the PMA, erector spinae muscle area or the pectoralis muscle area and, importantly, muscle area measurements were exclusively performed manually, which is probably inferior to the AI‐based approach used in our analysis.^19^, ^20^, ^22^, ^23^, ^24^, ^25^, ^26^

In our study, the postoperative 6MWD and quality of life were significantly lower in obese patients after 6 and 12 months when compared with normal‐weight individuals. However, this was not shown for sarcopenic patients. This might be due to other potential confounders as the right ventricular function or cardiopulmonary deconditioning. Interestingly, obese patients did not improve their walking distance within 6 months compared with the preoperative 6MWD, whereas the walking distance significantly improved in sarcopenic patients. Consistently, Vest et al. demonstrated a significant increase of muscle mass and fat mass within 6 months after LVAD implantation.^35^ Despite the benefit in exercise capacity, sarcopenic patients showed no improvement in the 6‐month quality of life assessment. Potentially confounding factors might be anxiety and depression, a lack of the social networks or supportive family environment leading to malnutrition behavioural tendencies and a loss of quality of life.

Overall, the comparability of the literature is limited by the use of various methods of body composition measures and the complexity of the LVAD cohort. Further limitations derive from the retrospective nature of this analysis that was performed in the largest, but still single, LVAD centre. The data were analysed only descriptively with a univariate regression analysis, generating hypotheses for further research. Women were underrepresented in this analysis, which is typical for LVAD studies and mainly due to the lower implantation rate in women.^11^, ^19^, ^38^, ^39^

In conclusion, AI‐derived body composition represents an accessible method that can be performed post‐processing with imaging data that derives from diagnostics that are routinely conducted in preparation for LVAD implantation. AI‐based body composition analyses provide a comprehensive survey of muscle and adipose tissue, allowing a precise characterization of preoperative risk factors. Adipose tissue appears to be the most important imaging‐derived body composition parameter associated with impaired outcome after LVAD implantation. The identification of patients at risk potentially leads to more individualized approaches in terms of ‘prehabilitation’, nutritive coaching, novel medical treatments and possibly perioperative anti‐infective concepts. In future, clinical trials investigating the effects of preoperative reduction of adipose tissue areas are desirable to further examine this hypothesis.

## Conflict of interest statement

IAJ received speaker honoraria from AstraZeneca GmbH and Abiomed outside of the submitted work. FS received institutional grants from Novartis and Abbott, non‐financial support from Medtronic and institutional fees (speaker honoraria) from Orion Pharma and AstraZeneca GmbH outside of the submitted work. LR received a doctoral scholarship from the German Heart Foundation during the conduct of the project FrailtyVAD‐Tx and reports share holdings of Allianz SE, Carl Zeiss Meditec AG, CompuGroup Medical SE & Co. KGaA, Evotec SE and Fresenius Medical Care AG & Co. KGaA outside of the submitted work. EP declares financial activities in proctoring and lecturing for Abiomed GmbH, Abbott GmbH & Co. KG and Medtronic and participates in the advisory board for FineHeart. All the other authors have nothing to declare related to this work.

## Supporting information


**Figure S1.** Availability of assessments of walk distance and quality of life in 6‐months and 12‐moths follow up. 6MWT six minute walk test; FU follow up visit; QOL quality of life assessment.

**Table S1.** Preoperative body composition.

**Table S2.** Association of preoperative risk factors and body composition biomarkers.

**Table S3.** Body composition and postoperative outcome.

**Table S4.** Body composition and 6MWD or QoL prior and post LVAD implantation.

**Table S5.** Preoperative patient characteristics at LVAD implantation for subgroup with both preoperative vs. 6‐months follow‐up assessments.
