# Supplementary material for: Artificial intelligence‐based analysis of body composition predicts outcome in patients receiving long‐term mechanical circulatory support
Source: J Cachexia Sarcopenia Muscle. 2023 Dec 26;15(1):270–80. doi: 10.1002/jcsm.13402 (PMC10834347; doi:10.1002/jcsm.13402)
Supplement: Supplementary file 1 — Figure S1. Availability of assessments of walk distance and quality of life in 6‐months and 12‐moths follow up. 6MWT six minute walk test; FU follow up visit; QOL quality of life assessment. [file JCSM-15-270-s003.docx]

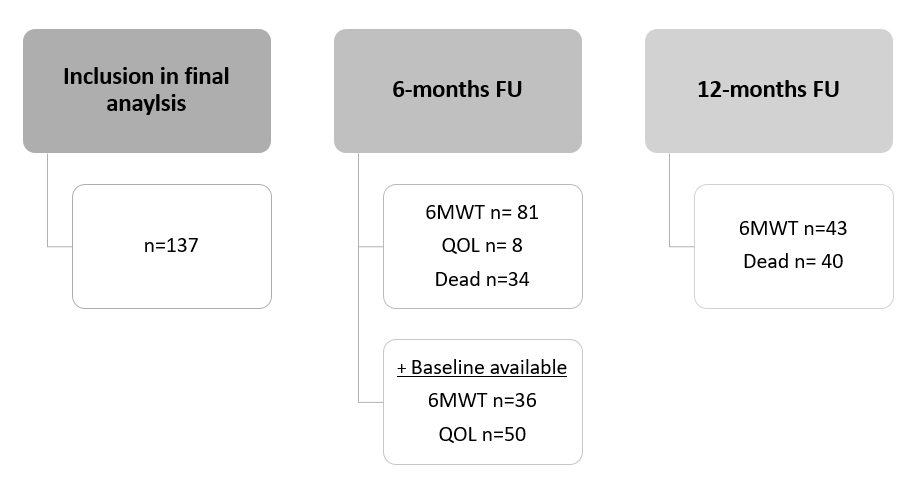


**Figure S1:** Availabliltiy of assessments of walk distance and quality of life in 6-months and 12-moths follow up. 6MWT six minute walk test; FU follow up visit; QOL quality of life assessment.
