# Supplementary material for: Artificial intelligence‐based analysis of body composition predicts outcome in patients receiving long‐term mechanical circulatory support
Source: J Cachexia Sarcopenia Muscle. 2023 Dec 26;15(1):270–80. doi: 10.1002/jcsm.13402 (PMC10834347; doi:10.1002/jcsm.13402)
Supplement: Supplementary file 2 — Table S1. Preoperative body composition. [file JCSM-15-270-s006.docx]

| **Table S1** Preoperative body composition | | | | |
| --- | --- | --- | --- | --- |
|  | **total n=137** | **male n=122** | **female n=15** | ***p*-value** |
| BMI in kg/m² | 28.21 ± 5.93 | 28.48 ± 5.57 | 24.15 [20.20; 27.68$]$ | 0.019 |
| BMI ≥ 30 kg/m² (%) | 44 (32.1) | 41 (33.6) | 3 (20.0) | 0.287 |
| VAT in cm² | 159.96 ± 115.61 | 170.48 ± 115.80 | 48.00 [20.01; 105;09] | 0.001 |
| SAT in cm² | 200.67 ± 118.97 | 200.21 ± 117.26 | 201.45 [105.27; 249.43] | 0.945 |
| ATR | 0.82 ± 0.46 | 0.88 ± 0.46 | 0.35 ± 0.15 | <0.001 |
| PMA in cm² | 17.76 ± 4.99 | 18.33 ± 4.93 | 13.16 ± 2.65 | <0.001 |
| TAMA in cm² | 143.55 ± 31.45 | 148.40 ± 29.33 | 104.11 ± 17.41 | <0.001 |
| LSMI in cm²/m² | 46.28 ± 9.97 | 47.20 ± 9.90 | 38.85 ± 7.21 | 0.001 |
| Sarcopenia (%) | 96 (70.1) | 88 (72.1) | 8 (53.3) | 0.134 |
| Sarcopenic obesity (%) | 34 (24.8) | 19 (15.6) | 0 | 0.100 |
| Continuous data were expressed as mean (±standard deviation); categorical data were expressed as number (%); skewed data as median [IQR]. **ATR** abdominal adipose tissue ratio; **BMI** body mass index; **LSMI** lumbar skeletal muscle index; **PMA** psoas muscle area; **SAT** subcutaneous adipose tissue; **TAMA** total abdominal muscle area; **VAT** visceral adipose tissue. | | | | |
