# Supplementary material for: Artificial intelligence‐based analysis of body composition predicts outcome in patients receiving long‐term mechanical circulatory support
Source: J Cachexia Sarcopenia Muscle. 2023 Dec 26;15(1):270–80. doi: 10.1002/jcsm.13402 (PMC10834347; doi:10.1002/jcsm.13402)
Supplement: Supplementary file 3 — Table S2. Association of preoperative risk factors and body composition biomarkers. [file JCSM-15-270-s005.docx]

| **Table S2** Association of preoperative risk factors and body composition biomarkers | | | | | | | |
| --- | --- | --- | --- | --- | --- | --- | --- |
| **Factor** | | **VAT** | **p-value** | **SAT** | **p-value** | **ATR** | **p-value** |
| **RV-EF** | **<30**  **n=24** | 162.78  [63.26, 236.50] | 0.861 | 148.58  [112.65, 207.51] | 0.145 | 0.92  [0.59, 1.14] | 0.260 |
|  | **>30**  **n=113** | 152.86  [54.91, 245.05] |  | 194.19  [116.57, 286.31] |  | 0.71  [0.45, 1.08] |  |
| **Nt-pro BNP** | **<8100**  **n=80** | 153.72 [55.06, 249.66] | 0.867 | 176.73 [114.30, 272.23] | 0.745 | 0.72 [0.43, 1.10] | 0.767 |
|  | **>8100**  **n=56** | 150.40 [60.55, 233.46] |  | 197.55 [115.23, 273.51] |  | 0.80 [0.49, 1.08] |  |
| **Creatinine** | **≤1.2**  **n=48** | 114.04  [36.70, 216.40] | 0.056 | 176.13  [104.33, 270.75] | 0.428 | 0.52  [0.40, 0.96] | 0.014 |
|  | **>1.2**  **n=88** | 160.00  [65.05, 261.08] |  | 194.25  [115.47, 281.02] |  | 0.84 [0.55, 1.12] |  |
| **6MWD** | **<200m**  **n=47** | 171.30  [71.88, 281.46] | 0.580 | 194.31  [127.38, 293.46] | 0.719 | 0.84  [0.49, 1.19] | 0.992 |
|  | **≥200m**  **n=30** | 157.18  [75.70, 246.82] |  | 200.18  [111.54, 291.52] |  | 0.81  [0.54, 1.16] |  |
| **EQ5D-5L** | **≤0.5**  **n=37** | 171.30  [105.09, 280.69] | 0.633 | 198.38  [137.98, 335.87] | 0.244 | 0.81  [0.48, 1.17] | 0.249 |
|  | **>0.5**  **n=28** | 160.14  [115.18, 249.85] |  | 201.02  [128.87, 258.54] |  | 0.87  [0.70, 1.18] |  |
| **Factor** | | **PMA** | **p-value** | **TAMA** | **p-value** | **LSMI** | **p-value** |
| **RV-EF** | **<30**  **n=24** | 18.63  [14.66, 20.72] | 0.374 | 141.34  [129.92, 156.88] | 0.726 | 47.30  [38.11, 2.94] | 0.946 |
|  | **>30**  **n=113** | 16.80  [13.89, 20.49] |  | 137.86  [122.37, 167.60] |  | 44.94  [40.43, 52.37] |  |
| **Nt-pro BNP** | **<8100**  **n=80** | 17.01  [14.34, 21.33] | 0.381 | 137.89  [125.43, 167.41] | 0.627 | 46.13  40.54, 53.18] | 0.379 |
|  | **>8100**  **n=56** | 16.84  [13.85, 19.98] |  | 139.20  [122.24, 162.64] |  | 44.81  [38.44, 52.36] |  |
| **Creatinine** | **≤1.2**  **n=48** | 16.40  [13.92, 20.30] | 0.590 | 137.64  [127.97, 168.24] | 0.761 | 46.23  [41.29, 4.52] | 0.259 |
|  | **>1.2**  **n=88** | 17.08  [14.29, 20.94] |  | 139.41  [119.53, 166.37] |  | 44.40  [38.31, 52.37] |  |
| **6MWD** | **<200m**  **n=47** | 17.08  [14.30, 20.75] | 0.722 | 145.09  [130.56, 168.44] | 0.646 | 47.82  [41.95, 54.58] | 0.465 |
|  | **≥200m**  **n=30** | 16.26  [14.19, 20.57] |  | 140.16  [128.71, 171.83] |  | 45.90  [40.72, 53.11] |  |
| **EQ5D-5L** | **≤0.5**  **n=37** | 17.05  [14.47, 20.80] | 0.711 | 137.41  [125.52, 168.84 | 0.368 | 47.04  [40.66, 54.80] | 0.926 |
|  | **>0.5**  **n=28** | 16.22  [14.30, 20.41] |  | 147.70  [130.74, 168.71 |  | 46.59  [41.89, 52.36] |  |
| Data is presented as median [IQR]. **6MWD** six minute walk distance; **ATR** abdominal adipose tissue ratio; **EQ5D**-**5L** quality of life assessment; **LSMI** lumbar skeletal muscle index; **NT-proBNP** N-terminal pro brain natriuretic peptide; **PMA** psoas muscle area; **RV-EF** right ventricular function; **SAT** subcutaneous adipose tissue; **TAMA** total abdominal muscle area; **VAT** visceral adipose tissue. | | | | | | | |
