# Supplementary material for: Artificial intelligence‐based analysis of body composition predicts outcome in patients receiving long‐term mechanical circulatory support
Source: J Cachexia Sarcopenia Muscle. 2023 Dec 26;15(1):270–80. doi: 10.1002/jcsm.13402 (PMC10834347; doi:10.1002/jcsm.13402)
Supplement: Supplementary file 4 — Table S3. Body composition and postoperative outcome. [file JCSM-15-270-s002.docx]

| **Table S3** Body composition and postoperative outcome | | | | | | | | | | | |
| --- | --- | --- | --- | --- | --- | --- | --- | --- | --- | --- | --- |
| **Outcome** |  | **Adipose tissue** | | | **Muscle tissue** | | | **Sarcopene** | | **Sarcopene obese** | |
|  |  | **VAT** | **SAT** | **ATR** | **PMA** | **TAMA** | **LSMI** | **Yes** | **No** | **Yes** | **No** |
| **in-hospital mortality** | yes  29 (21.2)  No  108 (78.8)  ***p*-value** | 168.11 [134.19, 285.27]  135.42  [49.44, 227.91]  **0.033** | 227.28 [139.38, 304.35]  173.81 [97.65, 254.16]  **0.009** | 0.81 [0.57, 1.02]  0.72 [0.426, 1.12]  **0.454** | 17.11 [14.73, 19.83]  16.84 [13.86, 21.23]  **0.916** | 141.07 [127.63, 168.04]  137.89  [121.98, 161.79]  **0.514** | 46.33 [42.00, 53.02]  44.67 [38.56, 52.78]  **0.190** | 20  (20.8)  76  (79.2)  **0.883** | 9  (22.0)  32  (78.0) | 6 (31.6)  13 (68.4)  **0.231** | 23 (19.5)  95 (80.5) |
| **12-months mortality** | Yes  40 (29.2)  No  97 (70.8)  ***p*-value** | 155.50 [63.62, 254.58]  151.60 [49.02, 233.68]  **0.498** | 187.45 [120.36, 300.96]  185.29 [103.29, 267.54]  **0.404** | 0.80 [0.56, 1.01]  0.73 [0.43, 1.13]  **0.474** | 17.05 [13.87, 21.38]  16.85 [13.91, 20.87]  **0.225** | 137.92 [121.52, 167.34]  137.41 [122.11, 161.46]  **1.000** | 44.90 [38.58, 52.98]  45.20 [39.57, 52.37]  **0.885** | 29 (30.2)  67 (69.8)  **0.690** | 11 (26.8)  30 (73.2) | 6 (31.6)  13 (68.4)  **0.806** | 34 (28.8)  84 (71.2) |
