# Supplementary material for: Artificial intelligence‐based analysis of body composition predicts outcome in patients receiving long‐term mechanical circulatory support
Source: J Cachexia Sarcopenia Muscle. 2023 Dec 26;15(1):270–80. doi: 10.1002/jcsm.13402 (PMC10834347; doi:10.1002/jcsm.13402)
Supplement: Supplementary file 5 — Table S4. Body composition and 6MWD or QoL prior and post LVAD implantation. [file JCSM-15-270-s004.docx]

| Table S4 Body composition and 6MWD or QoL prior and post LVAD implantation | | | |
| --- | --- | --- | --- |
|  | **Obese** | | ***p*-value** |
|  | **yes** | **no** |  |
| 6MWD preoperative in m | 244.84 ± 145.90 | 256.55 ± 124.59 | 0.764 |
| 6MWD after 6 months in m | 251.28 ± 166.00 | 370.19 ± 150.995 | **0.002** |
| QoL preoperative | 0.432 ± 0.312 | 0.519 ± 0.325 | 0.296 |
| QoL after 6 months | 0.560 ±0.311 | 0.695 ± 0.275 | **0.049** |
|  | **VAT ≥200 cm^2^** | | ***p*-value** |
|  | **yes** | **no** |  |
| 6MWD preoperative in m | 255.35 ± 123.16 | 249.93 ± 139.19 | 0.888 |
| 6MWD after 6 months in m | 282.15 ± 177.01 | 357.98 ± 153.64 | 0.051 |
| QoL preoperative | 0.452 ± 0.290 | 0.513 ± 0.341 | 0.459 |
| QoL after 6 months | 0.567 ± 0.322 | 0.692 ± 0.270 | 0.068 |
|  | **Sarcopene** | | **p-value** |
|  | **Yes** | **no** |  |
| 6MWD preoperative in m | 260.43 ± 121.04 | 232.67 ± 156.81 | 0.500 |
| 6MWD after 6 months in m | 347.84 ± 165.77 | 304.00 ± 159.77 | 0.263 |
| QoL preoperative | 0.520 ± 0.346 | 0.418 ± 0.247 | 0.240 |
| QoL after 6 months | 0.657 ± 0.303 | 0.638 ± 0.273 | 0.795 |
|  | **Sarcopene obese** | | ***p*-value** |
|  | **yes** | **no** |  |
| 6MWD preoperative in m | 266.75 ± 105.74 | 249.31 ± 137.05 | 0.735 |
| 6MWD after 6 months in m | 250.09 ± 153.80 | 346.93 ± 162.91 | 0.068 |
| QoL preoperative | 0.477 ± 0.385 | 0.490 ± 0.313 | 0.914 |
| QoL after 6 months | 0.550 ± 0.351 | 0.666 ± 0.282 | 0.222 |
| Data is presented as mean ± standard deviation. **6MWD** six minute walk distance; **QoL** quality of life; **VAT** visceral adipose tissue area. | | | |
