# Supplementary material for: Artificial intelligence‐based analysis of body composition predicts outcome in patients receiving long‐term mechanical circulatory support
Source: J Cachexia Sarcopenia Muscle. 2023 Dec 26;15(1):270–80. doi: 10.1002/jcsm.13402 (PMC10834347; doi:10.1002/jcsm.13402)
Supplement: Supplementary file 6 — Table S5. Preoperative patient characteristics at LVAD implantation for subgroup with both preoperative vs. 6‐months follow‐up assessments. [file JCSM-15-270-s001.docx]

| **Table S5** Preoperative patient characteristics at LVAD implantation for subgroup with both preoperative vs. 6-months follow-up assessments | | | | |
| --- | --- | --- | --- | --- |
|  | | **total**  **n=137** | **Subgroup 6MWT**  **n=36** | **Subgroup QoL**  **n=50** |
| Sex (%) |  |  |  |  |
|  | male | 122 (89.1) | 33 (91.7) | 46 (92.0) |
| Age in years |  | 58.21 ± 11.86 | 57.86 ± 10.34 | 57.66 ± 10.79 |
| Diagnosis (%) |  |  |  |  |
|  | DCM | 60 (43.8) | 15 (41.7) | 23 (46.0) |
|  | IHD | 72 (52.6) | 20 (55.6) | 26 (52.0) |
|  | others* | 5 (3.6) | 1 (2.8) | 1 (2.0) |
| Comorbidities (%) |  |  |  |  |
|  | diabetes mellitus | 26 (19.0) | 7 (19.4) | 8 (16.0) |
|  | COPD | 23 (16.8) | 4 (11.1) | 4 (8.0) |
| LV-EF in % |  | 18.85 ± 6.32 | 18.5 ± 6.62 | 19.8 ± 5.42 |
| LV-EDD in mm |  | 66.56 ± 11.46 | 64.74 ± 11.62 | 69.13 ± 10.21 |
| RV-EF in % |  | 41.02 ± 9.03 | 39.95 ± 9.67 | 42.04 ± 8.22 |
| VAD type (%) |  |  |  |  |
|  | HM3 | 38 (27.7) | 11 (30.6) | 16 (32.0) |
|  | HVAD | 98 (72.3) | 25 (69.4) | 34 (68.0) |
| INTERMACS profile (%) |  |  |  |  |
|  | I | 24 (18.8) | 2 (5.6) | 9 (18.0) |
|  | II | 43 (33.6) | 8 (22.2) | 15 (30.0) |
|  | III | 25 (19.5) | 10 (27.8) | 10 (20.0) |
|  | IV | 32 (25.0) | 15 (41.7) | 15 (30.0) |
|  | >IV | 5 (3.6) | 1 (2.8) | 1 (2.0) |
| MCS (%) |  |  |  |  |
|  | ECMO | 12 (8.8) | 1 (2.8) | 2 (4.0) |
|  | miniature axial flow pump | 9 (6.6) | 0 | 4 (8.0) |
|  | IABP | 1 (0.7) | 0 | 0 |
| RRT (%) |  | 13 (9.6) | 0 | 3 (6.0) |
| biomarkers |  |  |  |  |
|  | NTpro-BNP in pg/mL | 7056.0 [3585.0, 15363.0] | 9064.5 [3944.75, 15934.25.0] | 5032 [2714.0, 15939.0] |
|  | LDH U/L | 272.0 [217.5, 358.5] | 265.0 [208.5, 353.0] | 266.0 [210.5, 353.0] |
|  | Creatinine mg/dL | 1.5 [1.0, 1.8] | 1.4 [1.0, 1.8] | 1.3 [1.0, 1.8] |
|  | γGT U/L | 119.0 [69.75, 231.25] | 115.0 [68.5, 230.25] | 115.0 [69.0, 231.0] |
|  | Lactate mmol/L | 8.0 [6.0, 12.0] | 8 [6.0, 12.0] | 8.0 [6.0, 12.0] |
| Body composition | BMI in kg/m² | 28.21 ± 5.93 | 29.98 ± 6.86 | 29.58 ± 6.77 |
|  | VAT in cm² | 159.96 ± 115.61 | 187.81 ± 118.89 | 188.71 ± 108.67 |
|  | SAT in cm² | 200.67 ± 118.97 | 228.85 ±127.07 | 233.59 ± 126.289 |
|  | ATR | 0.82 ± 0.46 | 0.87 ± 0.50 | 0.87 ± 0.46 |
|  | PMA in cm² | 17.76 ± 4.99 | 18.05 ± 5.34 | 18.26 ± 5.11 |
|  | TAMA in cm² | 143.55 ± 31.45 | 148.51 ± 30.23 | 150.10 ± 28.91 |
|  | LSMI in cm²/m² | 46.28 ± 9.97 | 47.48 ± 10.33 | 48.0 ± 9.71 |
|  | Sarcopenia (%) | 96 (70.1) | 24 (66.7) | 33 (66.0) |
|  | Sarcopenic obesity (%) | 34 (24.8) | 7 (19.4) | 7 (14.0) |
| Continuous data were expressed as mean (±standard deviation); categorical data were expressed as number (%); skewed data as median (IQR). ATR abdominal adipose tissue ratio; BMI body mass index; COPD chronic obstructive pulmonary disease; DCM dilated cardiomyopathy; ECMO extracorporeal membrane oxygenation; EDD enddiastolic diameter; EF ejection fraction; HD heart disease; HM3 heart mate 3; HVAD heartware assist device; IABP intra-aortic balloon pump; IHD ischemic heart disease; INTERMACS interagency registry for mechanically assisted circulatory support; LDH lactate dehydrogenase; LSMI lumbar skeletal muscle index; LV left ventricle; MCS mechanical circulatory support; NTpro-BNP N-terminal prohormone of brain natriuretic peptide; PMA psoas muscle area; RRT renal replacement therapy; RV right ventricle; ; SAT subcutaneous adipose tissue; TAMA total abdominal muscle area; VAD ventricular assist device; VAT visceral adipose tissue; γGT γ-glutamyltransferase; 6MWD six-minute walk distance; *other: valvular heart disease (n=2), congenital heart disease (n=2/n=1); hypertrophic cardiomyopathy (n=1). | | | | |
